# Supplementary material for: A network analysis to identify mediators of germline-driven differences in breast cancer prognosis
Source: Nat Commun. 2020 Jan 16;11:312. doi: 10.1038/s41467-019-14100-6 (PMC6965101; doi:10.1038/s41467-019-14100-6)

Supplementary Information

A network analysis to identify mediators of germline-driven differences in breast cancer prognosis

Escala-Garcia *et al.*

Supplementary Table 1. Summary of invasive breast cancer cases, events and follow-up by genotyping study and ER-status. Details about the 12 studies are described elsewhere[4].

Study	All cases		ER-positive		ER-negative		Number of principal components*
	N (breast cancer deaths)	Person-years	N (breast cancer deaths)	Person-years	N (breast cancer deaths)	Person-years	
BCAC-OncoArray *comprising 61 BCAC studies	49,843 (2,826)	280,653	3,546 (1,640)	194,729	7,826 (881)	43,008	2
BCAC-COGS *comprising 38 BCAC studies	22,708 (1,302)	121,945	15,519 (740)	85,707	3,731 (391)	19,796	9
CGEMS	1,145 (93)	7,711	--	--	--	--	0
SASBAC	787 (69)	3,739	483 (44)	2,294	108 (9)	502	0
UK2	2,763 (233)	23,112	--	--			3
Metabric	369 (86)	1,570	291 (59)	1,268	63 (25)	225	1
PG-SNPs	1,786 (204)	5,820	1,188 (116)	3,916	586 (87)	1,888	2
HEBCS	742 (285)	4,666	492 (172)	3,458	196 (101)	982	0
SUCCESS-A	3,312 (175)	13,145	2,265 (83)	9,289	1,017 (90)	3,806	0
BPC3-CPSII	293 (30)	2,544	--	--	293 (30)	2,544	0
BPC3-EPIC	476 (74)	2,226	--	--	476 (74)	2,226	0
BPC3-NHS2	233 (36)	2,732	--	--	233 (36)	2,732	0
**Training set**	**84,457 (5,413)**		**55,701 (2,854)**		**14,529 (1,724)**		

BCAC: Breast Cancer Association Consortium, ER: estrogen receptor

Supplementary Table 2. Summary of invasive breast cancer cases, events and follow-up by genotyping array and ER-status for the independent set. Details about the study are described elsewhere[4].

	All cases		ER-positive		ER-negative	
	**N (breast cancer deaths)**	**Person-years**	**N (breast cancer deaths)**	**Person-years**	**N (breast cancer deaths)**	**Person-years**
BCAC-OncoArray *SEARCH study	3,723 (110)		2,691 (55)		408 (26)	
BCAC-COGS *SEARCH study	7,539 (1,010)		5,128 (561)		1,058 (215)	
**Independent set**	**12,381 (1,120)**	60,025	**7,819 (616)**	36,859	**1,466 (241)**	7,088

BCAC: Breast Cancer Association Consortium, ER: estrogen receptor

Supplementary Table 3. Variants and their coefficients included in the computation of the Polygenic Hazard Score (PHS) for each Germline-Related Prognostic Module. The variant identifiers have the format "<Chromosome>_<Build19Position>_<RefAllele>_<AltAllele>". All alleles are reported on the forward strand.

GRPM	Variant	Coefficients
G-alpha signaling events (I)	19_3086486_A_G	-0.1268
	19_3089773_T_C	-0.1011
G-alpha signaling events (II)	19_3081157_T_C	-0.1430
	19_3084795_A_G	-0.0599
	19_3089773_T_C	-0.0558
Circadian clock	1_7860276_AT_ATT	0.0793
	1_7870048_T_C	0.0827
	1_7915742_CATT_C	0.0849
	1_7918598_A_C	0.0426
	1_7924023_C_T	0.1232
	1_7927086_C_T	-0.2161
	1_7946161_C_T	0.1664
	12_56849340_C_G	-0.2372
	12_56856618_C_T	0.1022
	15_66666223_T_C	-0.1605
	17_8005118_C_T	0.0091
	17_8007650_T_C	0.1323
	17_8016373_T_G	-0.1230
	17_8055999_C_A	0.1506
Regulation of cell growth and angiogenesis	21_44031933_A_G	0.1715
	21_44244882_A_G	0.1314
	3_14105089_A_G	0.0806

	3_14158438_C_G<br>5_150837810_C_CAT	-0.2045<br>0.1310
Rho GTPases	15_80401077_GT_GTT<br>16_22346038_TG_T<br>17_43185500_G_A<br>17_43244700_A_C<br>17_43266487_G_A<br>19_14570329_C_CA<br>2_135748039_T_G<br>4_148757466_A_C<br>4_148946690_G_T<br>4_148949173_A_C<br>4_148970403_C_T	0.0926<br>-0.2491<br>-0.0972<br>0.0609<br>-0.0965<br>0.1035<br>0.1789<br>-0.1578<br>0.0551<br>0.0639<br>0.0596

Supplementary Table 4. P values obtained in the independent set for each high confidence Germline-Related Prognostic Module (GRPM)'s PHS: for the Estrogen Receptor (ER)-status group in which the GRPM was identified (in bold) versus the other ER-status group.

High-confidence GRPM	Independent set P value	
Identified in ER-negative tumors	ER-negative	ER-positive
G-alpha signaling events (I)	**0.008**	0.154
G-alpha signaling events (II)	**0.009**	0.171
Circadian clock	**0.030**	0.167
Regulation of cell growth and angiogenesis	**0.026**	0.145
Identified in ER-positive tumors		
Rho GTPases	0.763	**0.020**

Supplementary Figure 1. Module-level enrichment analyses for the Estrogen Receptor (ER)-negative (a-d) and ER-positive (e) high-confidence GRMPs. **(a)** G-alpha signaling (I). **(b)** G-alpha signaling (II). **(c)** Circadian clock. **(d)** Regulation of cell growth and angiogenesis. **(e)** Rho GTPases. Reactome annotations were used for the enrichment. The visualization was done using the Cytoscape app ClueGo. We selected pathways with a P value < 0.05 only. The enrichment of the nodes is represented within the node size and the functional groups are represented by the name of the most significant term in the group.

a

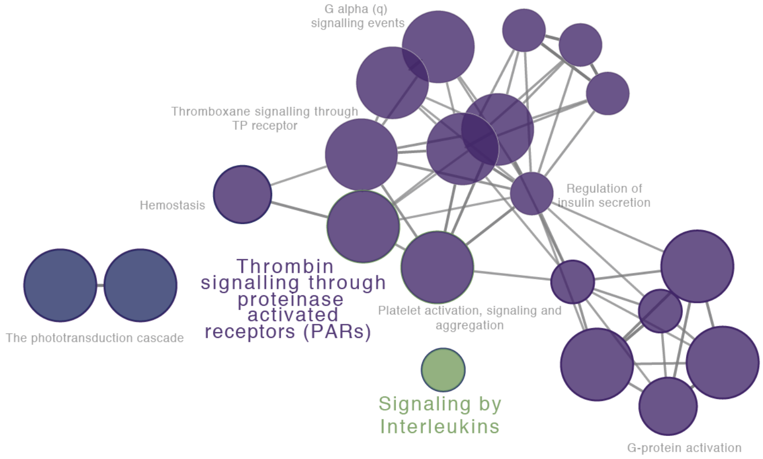

b

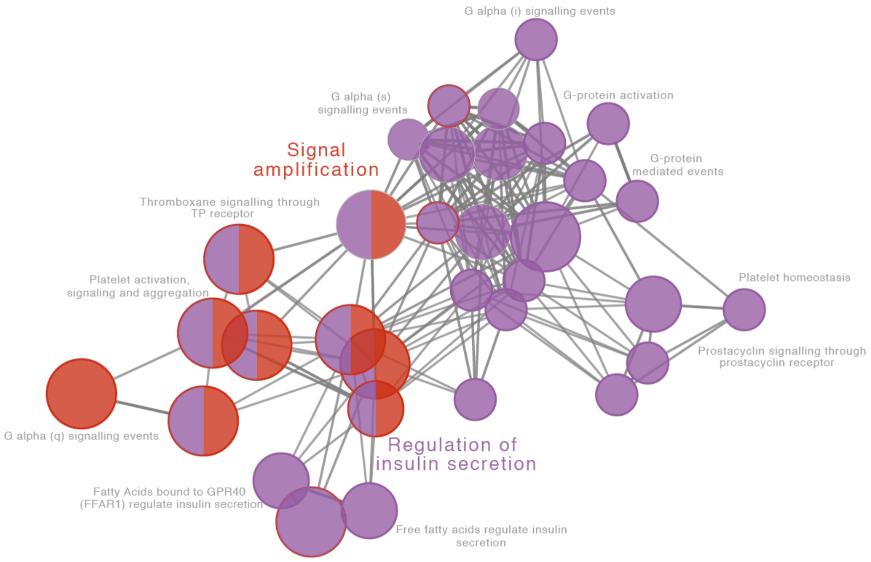

c

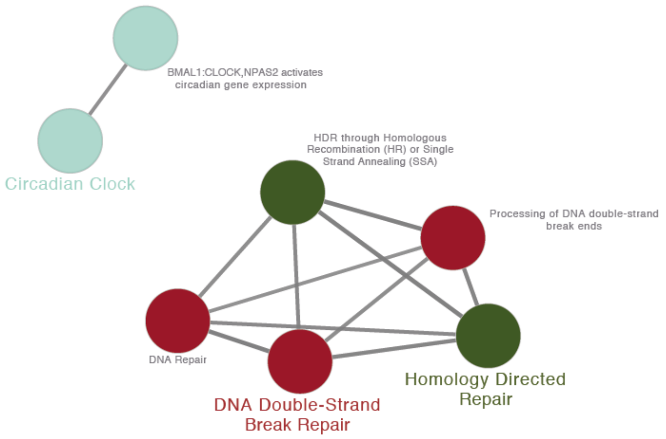
BMAL1:CLOCK,NPAS2 activates circadian gene expression
Circadian Clock
HDR through Homologous Recombination (HR) or Single Strand Annealing (SSA)
Processing of DNA double-strand break ends
DNA Repair
DNA Double-Strand Break Repair
Homology Directed Repair

d

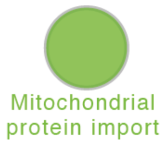
Mitochondrial protein import

e

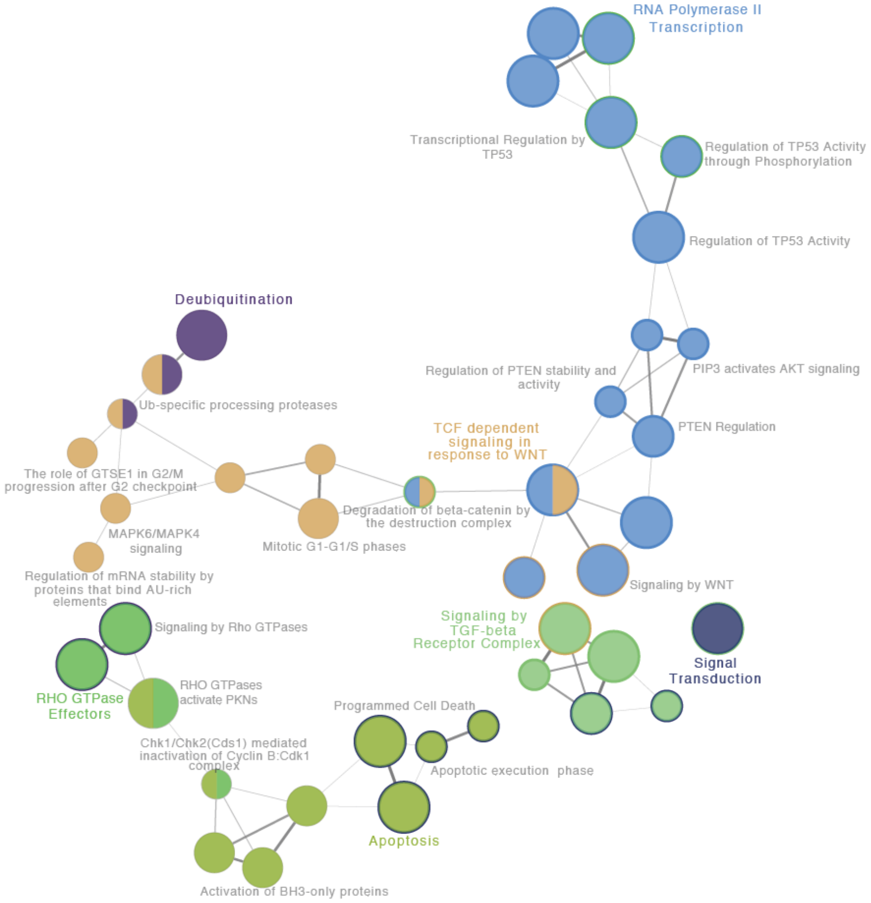
RNA Polymerase II Transcription
Transcriptional Regulation by TP53
Regulation of TP53 Activity through Phosphorylation
Regulation of TP53 Activity
Deubiquitination
Regulation of PTEN stability and activity
PIP3 activates AKT signaling
Ub-specific processing proteases
TCF dependent signaling in response to WNT
PTEN Regulation
The role of GTSE1 in G2/M progression after G2 checkpoint
Degradation of beta-catenin by the destruction complex
MAPK6/MAPK4 signaling
Mitotic G1-G1/S phases
Regulation of mRNA stability by proteins that bind AU-rich elements
Signaling by WNT
Signaling by Rho GTPases
Signaling by TGF-beta Receptor Complex
Signal Transduction
RHO GTPase Effectors
RHO GTPases activate PKNs
Programmed Cell Death
Chk1/Chk2(Cds1) mediated inactivation of Cyclin B:Cdk1 complex
Apoptotic execution phase
Apoptosis
Activation of BH3-only proteins

Supplementary Figure 2. Visualizations of the downstream enrichment analysis for the Reactome annotations for each high-confidence GRPM using the EnrichmentMap Cytoscape app. **(a)** G-alpha signaling (I). **(b)** G-alpha signaling (II). **(c)** Circadian clock. **(d)** Regulation of cell growth and angiogenesis. **(e)** Rho GTPases. Only biological processes with P value < 0.001 and False Discovery Rate (FDR) < 0.05 are shown in the representation. The colored circles represent gene sets, edges indicate overlapping genes, node size indicates the number of genes in the gene set and the color represents the associated FDR.

a

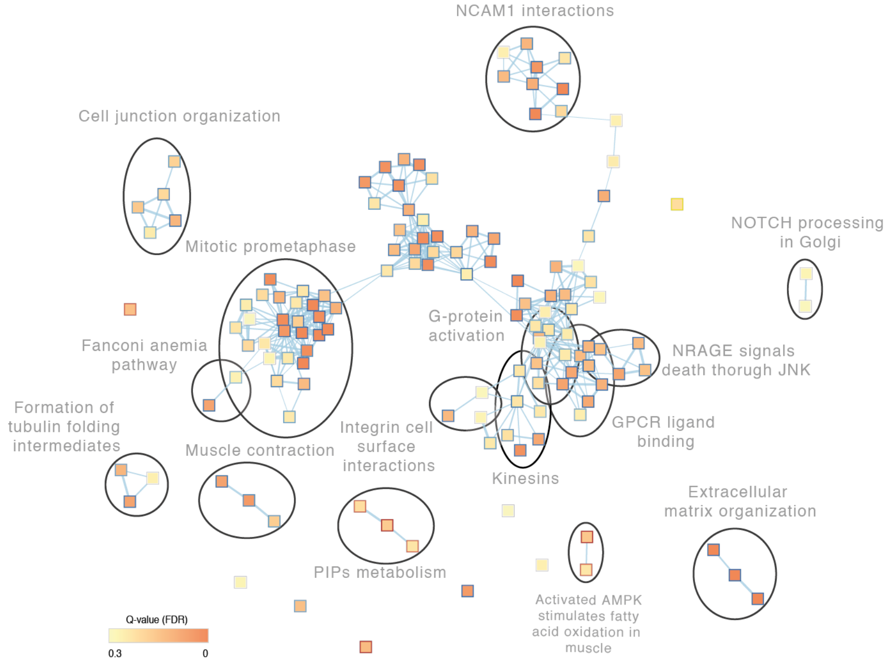

b

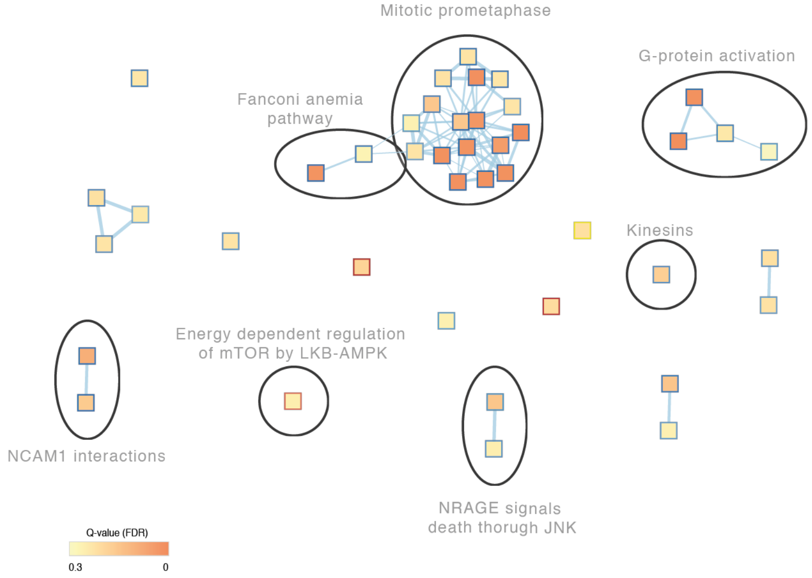

c

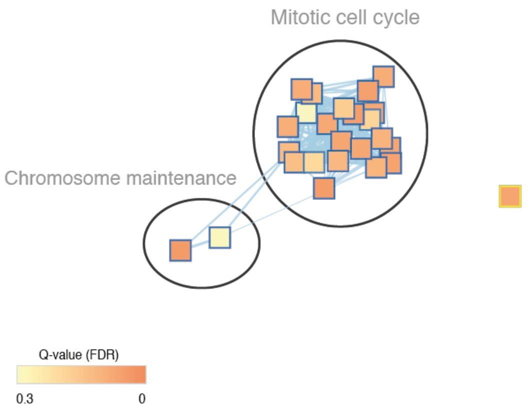
Mitotic cell cycle
Chromosome maintenance
Q-value (FDR)
0.3
0

d

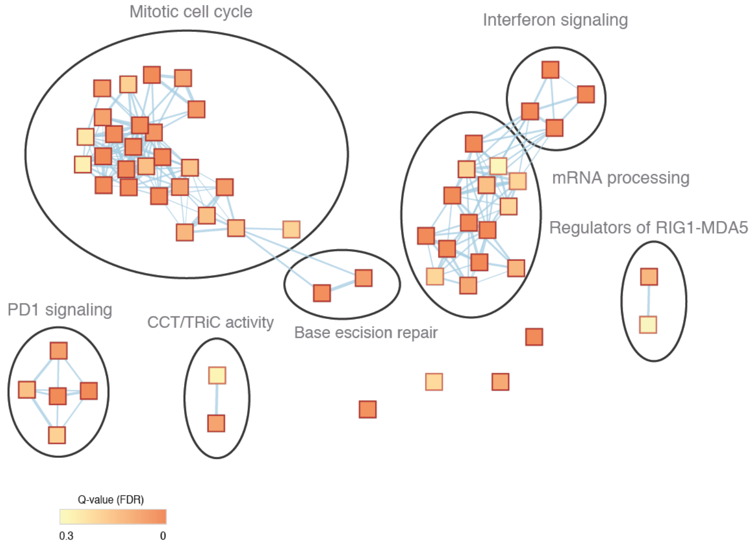
Mitotic cell cycle
Interferon signaling
mRNA processing
Regulators of RIG1-MDA5
PD1 signaling
CCT/TRiC activity
Base escision repair
Q-value (FDR)
0.3
0

e

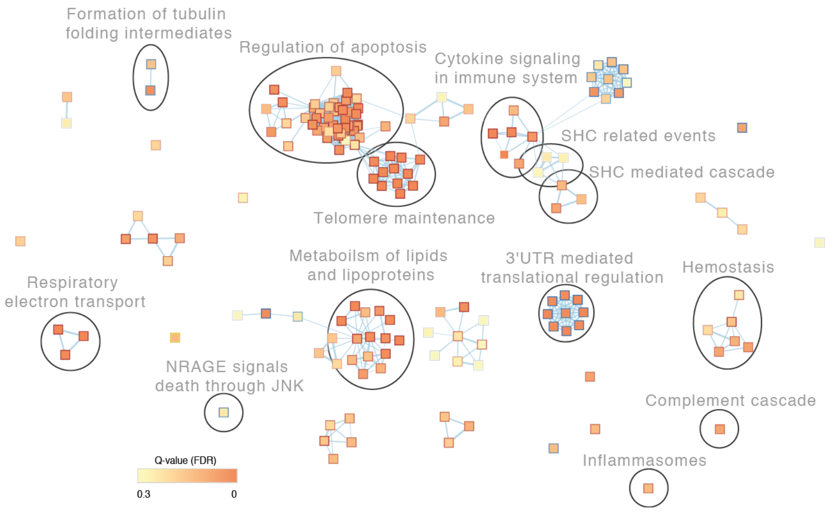
Formation of tubulin folding intermediates
Regulation of apoptosis
Cytokine signaling in immune system
SHC related events
SHC mediated cascade
Telomere maintenance
Respiratory electron transport
Metaboilsm of lipids and lipoproteins
3'UTR mediated translational regulation
Hemostasis
NRAGE signals death through JNK
Complement cascade
Inflammasomes
Q-value (FDR)
0.3
0

Supplementary Figure 3: Prognostic value of *GNA11* mRNA expression in Estrogen Receptor (ER)-negative breast tumors (n=1,214) with recurrence-free survival using KMplotter (kmplot.com/analysis). P value was computed using a logrank test. The Affymetrix IDs is 213766_x_at (*GNA11*).

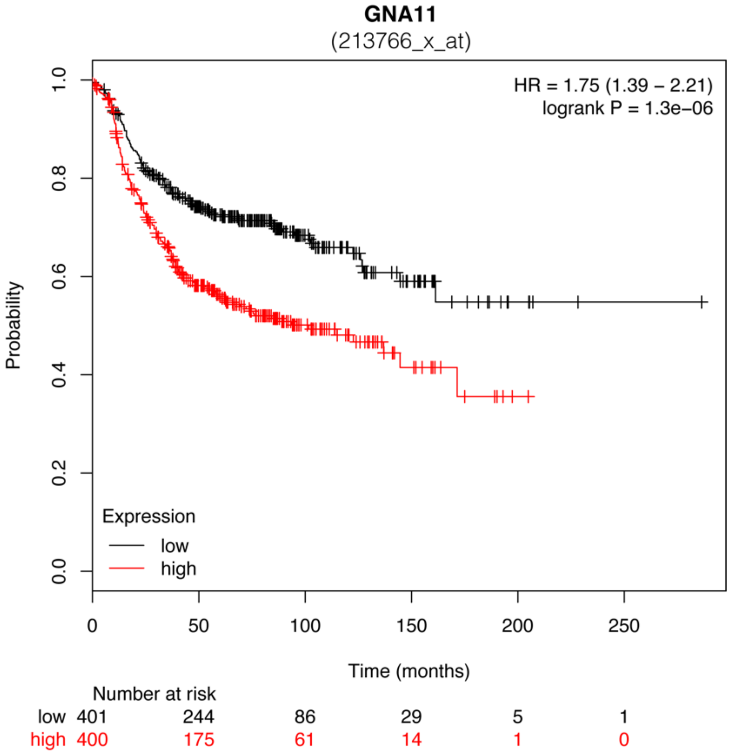

Supplementary Figure 4: Scatter plots showing the -log10 P value of the ~21,800 gene scores computed within a 50-kb window-size around the gene region. Each dot represents a gene score. The correlations shown are Pearson correlations. **(a)** Estrogen Receptor (ER)-positive vs all breast cancers. **(b)** ER-negative vs all breast cancers. **(c)** ER-negative vs ER-positive breast cancers.

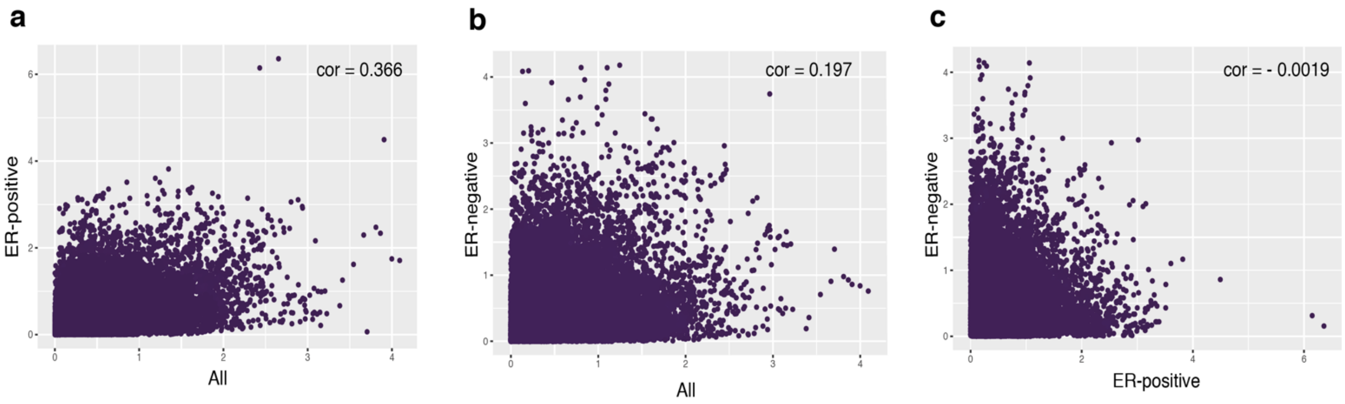

Supplementary Figure 5. Boxplots comparing the distributions of the Polygenic Hazard Scores (PHSs) for the Estrogen Receptor (ER)-status group in which the Germline-Related Prognostic Module (GRPM) was identified (red) versus the other ER-status (blue). The plot displays the median (center line), lower and upper hinges (25th and 75th percentiles respectively), two whiskers (scores outside the middle 50%) and all outlying points individually. **(a)** for the ER-negative high-confidence GRPMs. **(b)** for the ER-positive high-confidence GRPM. The Y-axes show the value of each PHS.

a

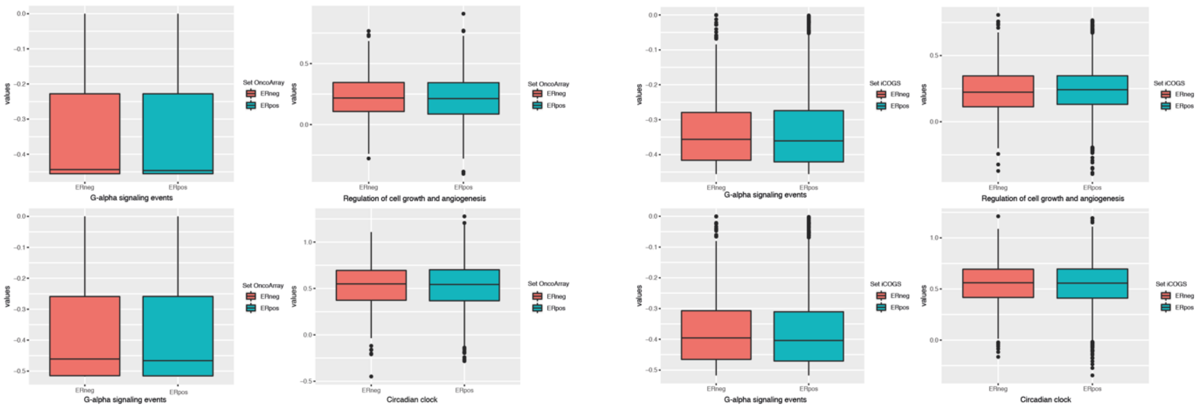

b

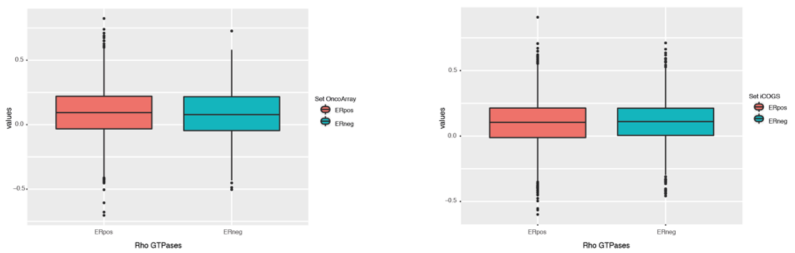

Supplementary Figure 6. QQ-plots of the observed and expected -log10 P values comparing Pascal's genes scores based on the maximum (left) and sum (right) statistics. **(a)** All breast cancer gene scores. **(b)** Estrogen Receptor (ER)-negative gene scores. **(c)** ER-positive gene scores.

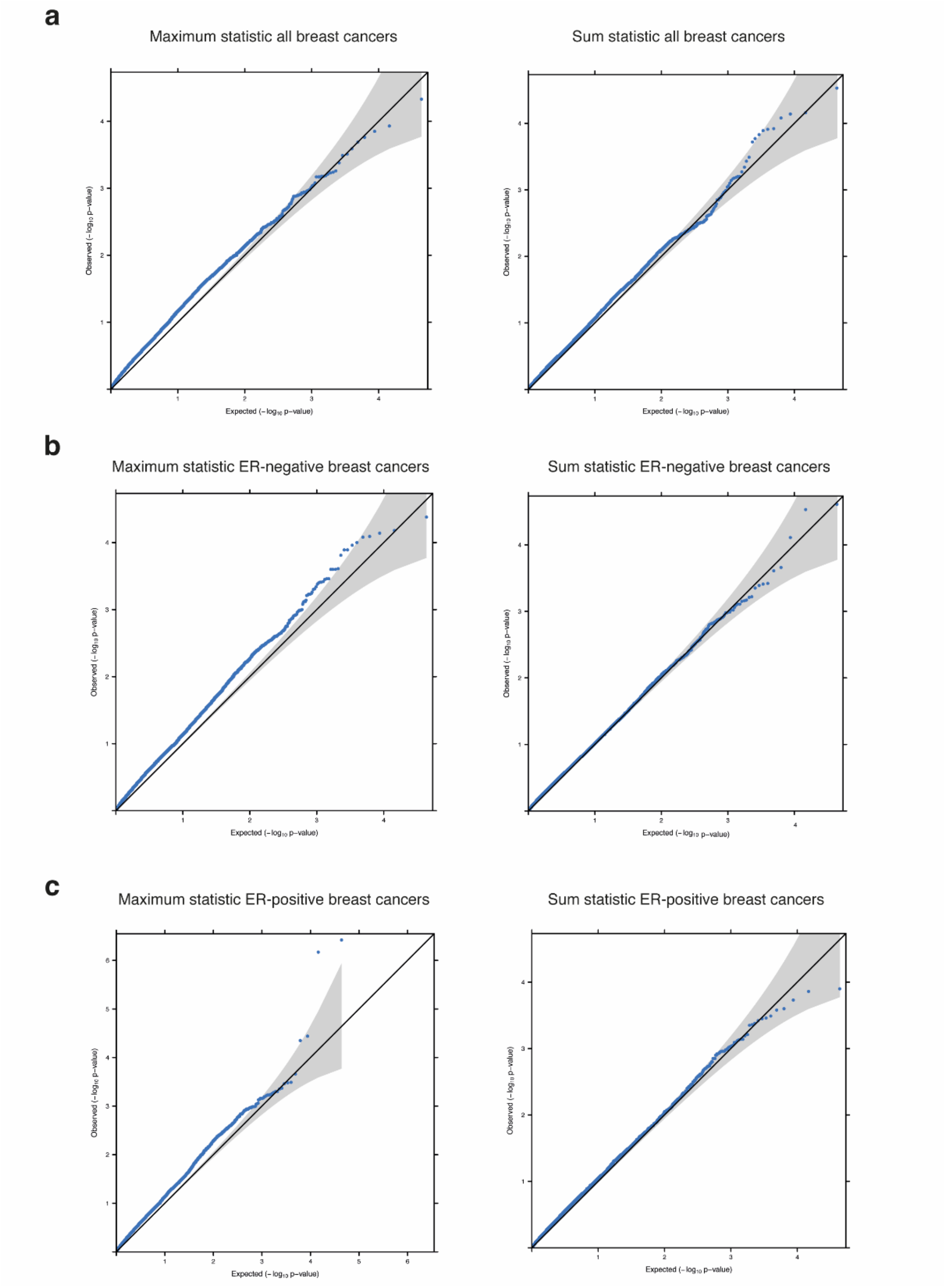

Supplement: Supplementary file 2 — Supplementary Information [file 41467_2019_14100_MOESM2_ESM.pdf]
