# Supplementary material for: A network analysis to identify mediators of germline-driven differences in breast cancer prognosis
Source: Nat Commun. 2020 Jan 16;11:312. doi: 10.1038/s41467-019-14100-6 (PMC6965101; doi:10.1038/s41467-019-14100-6)
Supplement: Supplementary file 3 — Description of Additional Supplementary Files [file 41467_2019_14100_MOESM3_ESM.pdf]

Description of Additional Supplementary Files

File Name: Supplementary Data 1
Description: List of all significant HotNet2 prognostic modules identified using the proteinprotein interaction network IrefIndex for Estrogen Receptor (ER)-positive and for ER-negative breast cancer.

File Name: Supplementary Data 2
Description: Positively and negatively enriched pathways identified in the downstream analyses for the MSigDB Hallmark gene sets and for Reactome gene sets for the Estrogen Receptor (ER)- negative Germline-related Prognostic Module G-alpha signaling (I).

File Name: Supplementary Data 3
Description: Positively and negatively enriched pathways identified in the downstream analyses for the MSigDB Hallmark gene sets and for Reactome gene sets for the Estrogen Receptor (ER)- negative Germline-related Prognostic Module G-alpha signaling (II).

File Name: Supplementary Data 4
Description: Positively and negatively enriched pathways identified in the downstream analyses for the MSigDB Hallmark gene sets and for Reactome gene sets for the Estrogen Receptor (ER)- negative Germline-related Prognostic Module Circadian clock.

File Name: Supplementary Data 5
Description: Positively and negatively enriched pathways identified in the downstream analyses for the MSigDB Hallmark gene sets and for Reactome gene sets for the Estrogen Receptor (ER)- negative Germline-related Prognostic Module Regulators of cell growth and angiogenesis.

File Name: Supplementary Data 6
Description: Positively and negatively enriched pathways identified in the downstream analyses for the MSigDB Hallmark gene sets and for Reactome gene sets for the Estrogen Receptor (ER)- positive Germline-related Prognostic Rho-GTPases.
