# Supplementary material for: A network analysis to identify mediators of germline-driven differences in breast cancer prognosis
Source: Nat Commun. 2020 Jan 16;11:312. doi: 10.1038/s41467-019-14100-6 (PMC6965101; doi:10.1038/s41467-019-14100-6)
Supplement: Supplementary file 10 — Reporting Summary [file 41467_2019_14100_MOESM10_ESM.pdf]

nature research

Corresponding author(s): Sander Canisius and Marjanka K. Schmidt
Last updated by author(s): Dec 10, 2019

Reporting Summary

Nature Research wishes to improve the reproducibility of the work that we publish. This form provides structure for consistency and transparency in reporting. For further information on Nature Research policies, see Authors & Referees and the Editorial Policy Checklist.

Statistics

For all statistical analyses, confirm that the following items are present in the figure legend, table legend, main text, or Methods section.

n/a	Confirmed	
☐	☒	The exact sample size (*n*) for each experimental group/condition, given as a discrete number and unit of measurement
☐	☒	A statement on whether measurements were taken from distinct samples or whether the same sample was measured repeatedly
☐	☒	The statistical test(s) used AND whether they are one- or two-sided<br>*Only common tests should be described solely by name; describe more complex techniques in the Methods section.*
☐	☒	A description of all covariates tested
☐	☒	A description of any assumptions or corrections, such as tests of normality and adjustment for multiple comparisons
☐	☒	A full description of the statistical parameters including central tendency (e.g. means) or other basic estimates (e.g. regression coefficient) AND variation (e.g. standard deviation) or associated estimates of uncertainty (e.g. confidence intervals)
☐	☒	For null hypothesis testing, the test statistic (e.g. *F*, *t*, *r*) with confidence intervals, effect sizes, degrees of freedom and *P* value noted<br>*Give P values as exact values whenever suitable.*
☒	☐	For Bayesian analysis, information on the choice of priors and Markov chain Monte Carlo settings
☒	☐	For hierarchical and complex designs, identification of the appropriate level for tests and full reporting of outcomes
☐	☒	Estimates of effect sizes (e.g. Cohen's *d*, Pearson's *r*), indicating how they were calculated

Our web collection on statistics for biologists contains articles on many of the points above.

Software and code

Policy information about availability of computer code

Data collection	SQL database
Data analysis	HotNet2, Pascal, Cytoscape (version 3.4.0), R, glmnet (R package version 2.0-10), ShapeIT2, IMPUTE v2

For manuscripts utilizing custom algorithms or software that are central to the research but not yet described in published literature, software must be made available to editors/reviewers. We strongly encourage code deposition in a community repository (e.g. GitHub). See the Nature Research guidelines for submitting code & software for further information.

Data

Policy information about availability of data

All manuscripts must include a data availability statement. This statement should provide the following information, where applicable:
- Accession codes, unique identifiers, or web links for publicly available datasets
- A list of figures that have associated raw data
- A description of any restrictions on data availability

All 10-year breast cancer-specific survival summary estimates are available via the BCAC website (http://bcac.ccge.medschl.cam.ac.uk/bcacdata/). Individual patient data will not be made publicly available without request due to restraints imposed by the ethics committees of individual studies. Formal request can be made via the Data Access Coordination Committee (DACC) of BCAC (http://bcac.ccge.medschl.cam.ac.uk/). A subset of the data that supports the findings of this analysis is available at https://portal.gdc.cancer.gov/ (accession number phs000178).

Field-specific reporting

Please select the one below that is the best fit for your research. If you are not sure, read the appropriate sections before making your selection.

☒ Life sciences ☐ Behavioural & social sciences ☐ Ecological, evolutionary & environmental sciences

For a reference copy of the document with all sections, see nature.com/documents/nr-reporting-summary-flat.pdf

Life sciences study design

All studies must disclose on these points even when the disclosure is negative.

Sample size	The dataset included essentially all available GWAS data on breast cancer patients (84,457 breast cancer patients in total).
Data exclusions	We excluded samples and variants according to predefined filtering criteria to remove unreliable genotype calls as described in the methods. Patients exclusion included patients with metastasis at diagnosis and samples other than European ancestry.
Replication	12,381 independent patients were used for validation of the results.
Randomization	This was an observational genetic association study, therefore randomization was not relevant.
Blinding	The laboratories conducting the genotyping did not have access to phenotypic data (i.e. blinded). Genotype and phenotype were only combined during the computational analyses.

Reporting for specific materials, systems and methods

We require information from authors about some types of materials, experimental systems and methods used in many studies. Here, indicate whether each material, system or method listed is relevant to your study. If you are not sure if a list item applies to your research, read the appropriate section before selecting a response.

Materials & experimental systems

n/a	Involved in the study	
☒	☐	Antibodies
☒	☐	Eukaryotic cell lines
☒	☐	Palaeontology
☒	☐	Animals and other organisms
☐	☒	Human research participants
☒	☐	Clinical data

Methods

n/a	Involved in the study	
☒	☐	ChIP-seq
☒	☐	Flow cytometry
☒	☐	MRI-based neuroimaging

Human research participants

Policy information about studies involving human research participants

Population characteristics	Female patients with invasive breast cancer diagnosed at age>18 years, of European ancestry and with follow-up data available were included in the analyses. BCAC data from freeze 10 was used. The data included patients from 12 different cohorts that were genotyped by a variety of arrays providing genome-wide coverage of common variants. Analyses were based on variants that were imputed with imputation r2>0.8 and had minor allele frequency (MAF)>0.05.
Recruitment	Patients were recruited at different times before or after diagnosis, time under observation was calculated from the recruitment date (left truncation) in order to avoid possible bias produced by prevalent cases.
Ethics oversight	The study was performed in accordance with the Declaration of Helsinki. All individual studies, from which data were used, were approved by the appropriate medical ethical committees and/or institutional review boards. All study participants provided informed consent.

Note that full information on the approval of the study protocol must also be provided in the manuscript.
